# 人工智能辅助血细胞形态学检查的技术要求及其临床应用中国专家共识（2024年版）

**DOI:** 10.3760/cma.j.cn121090-20240217-00064

**Published:** 2024-04

**Authors:** 中华医学会血液学分会实验诊断学组

**Keywords:** 血细胞形态学检查, 人工智能, 临床应用, 数据管理与信息安全, 细胞识别谱, Blood cell morphology examination, Artificial intelligence, Clinical application, Data management and information security, Cell recognition spectrum

## Abstract

血细胞形态学检查是血液疾病诊断的重要手段，传统人工镜检存在效率低、易受主观影响等问题。人工智能技术的应用提高了血细胞检查的效率和质量，促进了检测结果的标准化。目前，多种人工智能设备已在临床使用或研究中，但其技术要求和配置各异。中华医学会血液学分会实验诊断学组组织相关专家，制订了本共识。本共识内容包括术语定义、适用范围、技术要求、临床应用、数据管理和信息安全，强调了标本制备、图像采集、图像分割算法、细胞特征提取与识别分类的重要性，并提出了细胞识别谱的基本要求。同时，对病理细胞的细分类、细胞训练与测试的要求、质量控制标准、辅助人工出具诊断报告等方面进行了详细说明。此外，还强调了数据管理和信息安全的重要性，确保患者信息的安全和数据的准确性。

## Abstract (en)

Blood cell morphological examination is a crucial method for the diagnosis of blood diseases, but traditional manual microscopy is characterized by low efficiency and susceptibility to subjective biases. The application of artificial intelligence (AI) technology has improved the efficiency and quality of blood cell examinations and facilitated the standardization of test results. Currently, a variety of AI devices are either in clinical use or under research, with diverse technical requirements and configurations. The Experimental Diagnostic Study Group of the Hematology Branch of the Chinese Medical Association has organized a panel of experts to formulate this consensus. The consensus covers term definitions, scope of application, technical requirements, clinical application, data management, and information security. It emphasizes the importance of specimen preparation, image acquisition, image segmentation algorithms, and cell feature extraction and classification, and sets forth basic requirements for the cell recognition spectrum. Moreover, it provides detailed explanations regarding the fine classification of pathological cells, requirements for cell training and testing, quality control standards, and assistance in issuing diagnostic reports by humans. Additionally, the consensus underscores the significance of data management and information security to ensure the safety of patient information and the accuracy of data.

血细胞形态学检查是血液系统疾病诊断中快速直观、经济实用的重要手段。传统的人工镜检耗时费力，对专业人员的技术、经验与基础理论要求较高，检测结果易受主观因素影响，难以批量化、标准化[1]。近年来，随着计算机技术的进步，人工智能在医疗领域的应用与日俱增，为提升个体化诊疗与实现精准医学提供强有力支撑[2]。血细胞形态学检查引入人工智能可大大降低人力成本，提高工作效率和质量，更易实现标准化，提高检测结果的一致性和可比性[3]–[5]。目前，国内外有多种人工智能辅助识别血细胞的相关设备进入临床使用或处于临床前研究阶段[6]–[13]，但其配置与技术要求各异。为了进一步解决人工智能图像识别技术在血细胞形态学检查临床应用中的实际问题，推动其使用标准化、规范化，创建和优化应用场景、学习模型及报告形式，中华医学会血液学分会实验诊断学组组织相关专家制定了本共识。

一、术语及适用范围

（一）术语

1. 人工智能（Artificial intelligence）：通常指通过普通计算机程序来呈现人类智能的技术，它表现出与人类智能（如推理和学习）相关的各种功能单元的能力[14]。

2. 数据集（Data set）：数据记录汇聚的数据形式[14]。

3. 训练集（Training set）：用于训练人工智能算法的数据集[14]。

4. 测试集（Testing set）：用于测试人工智能算法性能的数据集[14]。

5. 卷积神经网络（Convolutional Neural Network, CNN）：是一种直接从数据中学习的深度学习网络架构[15]。

6. 无极缩放（Infinite scale）：指无限平滑扩大缩小图片，在计算机图像处理和计算机图形学中，图像缩放是指对数字图像的大小进行调整的过程[16]。

7. 数字全息（Digital holography）：使用光电传感器件，例如电荷耦合元件（Charge-coupled device, CCD）、互补金属氧化物半导体（Complementary metal oxide semiconductor, CMOS）记录全息图，然后将全息图存入计算机，用计算机模拟光学衍射过程来实现被记录物体的全息再现和处理。

8. 每英寸像素点数（Dots Per Inch, DPI）：指每一英寸长度中，取样、可显示或输出点的数目，是一个用于点阵数码影像的量度单位。

9. 准确率（Accuracy）：指被正确分类的血细胞数量与血细胞总数之比[17]。

10. 精确率（Precision）：亦称查准率，指被正确分类为某一类型的血细胞数量与被分类为该类型的血细胞数量之比[17]。

11. 召回率（Recall）：亦称查全率，指被正确分类为某一类型的血细胞数量与应当被分类为该类型的血细胞数量之比[17]。


$$准确率=\frac{人工智能分类正确的血细胞数}{血细胞总数}=\frac{\sum_{i=1}^{m}n_{i,i}}{\sum_{i=1}^{m}\sum_{j=1}^{m}n_{i,j}}$$

$$精确率=\frac{x类人工智能分类正确的血细胞数}{人工智能分类为x类血细胞数之和}=\frac{n_{x,x}}{\sum_{i=1}^{m}n_{i,x}}$$

$$召回率=\frac{x类人工智能分类正确的血细胞数}{标注为x类血细胞数之和}=\frac{n_{x,x}}{\sum_{j=1}^{m}n_{x,j}}$$

m表示要区分的类别，n_ij_表示标注为i类、分类为j类的血细胞数，I, j=1、2、......、m

12. 血细胞识别谱（Hemocytes morphology profiles）：指纳入人工智能细胞识别范围的血细胞种类、系统所编制成的表册或所绘制成的图表。

13. 预分类（Pre-classification）：指预先按照种类、等级或性质分别归类。

（二）适用范围

使用人工智能设备辅助外周血、骨髓血细胞形态学检查的各级医疗机构、科研机构、生产商和教学单位可参照本共识。

二、人工智能辅助血细胞形态学检查的相关技术要求

（一）标本制备与染色

涂片与染色可选择手工或自动化操作。自动涂片制备机一般依据添加的样本量、推片速度、推片角度等参数制备血涂片和（或）骨髓涂片，相比于人工制片方式效率更高、稳定性更好[18]–[19]。自动涂片染色机可模拟手工染色过程，快速高效地染色大量样本，提高结果的可重复性和准确性。

人工智能辅助血细胞形态学检查的制片和染色质量要求：涂片应厚薄均匀，呈“舌形”，长度1.5～3.0 cm，分头、体、尾三部分，边缘整齐，两侧留有空隙，片头片尾无细胞凝集现象[20]。染色使用瑞氏-姬姆萨或瑞氏染色液，细胞形态要完整，着色要均匀、色彩鲜明、不偏色，无沉渣或染液结晶[21]。

（二）细胞图像采集

手动图像采集方法：手动聚焦，使用数码相机（CCD或CMOS）进行单视野细胞图片采集。

自动图像扫描采集方法：使用自动扫描采集系统，利用低倍镜（10×）快速扫描整张载玻片，通过无极缩放（物镜1×～100×）或固定放大倍数（如物镜20×、40×、100×）方式获取图像[10],[22]–[23]。图像采集应在血细胞涂片的体尾交界部采用连续性、城垛式的方式进行。

为了更好地获得数字图像，细胞自动扫描采集系统通常具备以下基本功能：

1. 数字化扫描：实现数字全息、高清晰、标准化的图像采集。物镜低放大倍数条件下实现全玻片扫描。

2. 变焦功能：至少输出200倍放大。

3. 图像质量：在100×光学放大倍数下，DPI>2.54×10^5^（0.1 µm），细胞图片亮度在10^3^～10^4^ cd/m^2^。肉眼观察图像清晰、光照均匀、对比度高，细胞边缘界限明了，细胞结构清楚（包括染色质、核仁、颗粒），能辨识单核细胞胞质细小颗粒和巨核细胞胞质云雾状颗粒。

4. 存储要求：具有临时和永久存储图片的功能，建议采用PNG或JPEG图像文件存储格式。可用存储空间在4 TB以上，具备数据冗余备份，传输速度80 MB以上，可滚动存储至少1 000张图像。

（三）细胞图像分割算法

图像分割是指将图像划分成若干个特定的、具有独特性质的区域并提出感兴趣目标的技术和过程，是图像处理与图像分析的关键步骤。血细胞图像分割需要将不同类型的细胞或有核细胞的核与质从图像中分离出来，以便精准地测量血细胞相关的各种参数。在深度学习技术兴起前，计算机领域的研究者们常使用传统图像处理方法（如基于阈值、边缘、区域、聚类、图论及特定理论等图像分割方法），但存在分割效果差、鲁棒性差等缺点[24]。随着计算技术的快速发展，深度学习图像分割方法通过其强大的学习能力，能够更好地捕捉图像中的语义信息，提高图像分割的准确性，被广泛应用于图像处理领域，例如SegNet[25]、DeepLab[26]、Mask R-CNN[27]、U-Net[28]、SAM[29]等。

（四）细胞特征提取与识别分类

细胞图像特征提取与识别分类是人工智能辅助血细胞形态学检查最核心的部分。细胞图像特征主要有细胞形态学特征（如边界周长、区域面积、紧凑性、矩形度、细胞核的分叶数、胞质中颗粒数、胞核中核仁数）、颜色特征（如红色、绿色、蓝色）、纹理特征（如熵、能量、惯性、局部同一性）等[30]–[34]。在细胞特征提取与识别分类中，采用的传统机器学习方法有主成分分析、支持向量机、模糊 C 均值聚类等[34]。不同于传统方法，深度学习方法（如以卷积神经网络为基础的深度学习算法、Transformer算法）更依赖于数据本身，经过大量图像数据的训练后能自动学习出不同图像的特征，并进行特征提取、识别分类等任务，已成为目前分析视觉图像的主要技术，常用算法有VggNet[35]、GoogLeNet[36]、ResNet[37]、YOLO[38]、MobileNet[39]、SSD[40]、R-CNN[41]、RetinaNet[42]、Vision Transformer[43]、DETR[44]等。这些算法可单独或组合应用，以寻找最优方案。训练集和测试集可用于算法的训练与测试[4],[30]。

（五）细胞的名称

对人工智能来说，细胞识别分类不具备区分病理细胞的能力。例如一种形态特征的细胞可能有多种病理意义，一种病理意义的细胞可能对应多种形态特征，但人工智能识别依据的是细胞的几何、颜色和纹理等细胞图像特征，只能对某种形态相似的细胞进行识别归类。如果按病理属性来分类识别细胞，不顾及细胞间形态的巨大差异，会导致识别准确率降低。因此，建议人工智能辅助识别的细胞名称应遵循形态学定义原则，尽可能采用传统命名来定义细胞的名称（部分未命名的异常细胞仍需要采用形态描述），并参考现有行业指南或建议的命名[45]–[47]。

（六）细胞识别谱

人工智能辅助细胞图像识别分类技术应是不断发展的以适应多元化的临床需求，但由于其尚在临床应用的初期阶段，为了满足临床实验诊断与血细胞分类指南的要求，应构建基本的细胞识别谱。人工智能辅助识别外周血细胞时，至少纳入常见的48种细胞，包括23种白细胞、17种成熟红细胞和8种血小板，以构成外周血细胞识别谱（表1）[45],[47]，同时能区别涂抹细胞和染料杂质。在识别骨髓有核细胞初期，细胞种类至少纳入50种细胞，包括常见的各阶段的造血细胞（粒细胞、红细胞、巨核细胞）、淋巴细胞、单核细胞、浆细胞及相应的病变细胞等，以构成骨髓细胞识别谱（表2）[48]–[51]。

**表1:** 外周血细胞识别谱

类别	系统	系列	幼稚阶段	成熟阶段	异常形态细胞
应纳入类别					
无核细胞	红细胞			成熟红细胞	大红细胞、小红细胞、低色素性红细胞、嗜多色性红细胞、椭圆形和卵圆形红细胞、泪滴形红细胞、球形红细胞、靶形红细胞、口形红细胞、棘形红细胞、裂片红细胞、点彩红细胞、皱缩红细胞、红细胞（含Howell-Jolly小体）、红细胞（含微生物寄生虫）、缗钱状排列红细胞
	血小板			散在血小板、成簇血小板	巨大血小板、大血小板、小血小板、少颗粒血小板、异形血小板、血小板聚集成堆
有核细胞	粒细胞	中性粒细胞	早幼粒细胞、中性中幼粒细胞、中性晚幼粒细胞	中性杆状核粒细胞、中性分叶核粒细胞	异常早幼粒细胞、Pelger-Huët畸形粒细胞
		嗜酸性粒细胞	未成熟嗜酸性粒细胞^a^	成熟嗜酸性粒细胞	异常嗜酸性粒细胞
		嗜碱性粒细胞	未成熟嗜碱性粒细胞^b^	成熟嗜碱性粒细胞	异常嗜碱性粒细胞
	淋巴细胞		幼稚淋巴细胞	成熟淋巴细胞	异常淋巴细胞或不典型改变的淋巴细胞（怀疑肿瘤性）、异型淋巴细胞或不典型改变的淋巴细胞（怀疑反应性）
	单核细胞		幼稚单核细胞	成熟单核细胞	异常单核细胞或不典型改变的单核细胞
	其他细胞				原始细胞、幼稚红细胞^c^、浆细胞
后期需纳入类别					
无核细胞	红细胞				其他异形红细胞
	血小板				其他异常血小板
有核细胞	粒细胞				多分叶核粒细胞、粒系巨幼变细胞、粒系巨幼样变细胞
	其他细胞				巨核细胞、吞噬细胞、内皮细胞、退化细胞、肥大细胞、分类不明细胞

**注** ^a^ 指中幼、晚幼嗜酸性粒细胞；^b^ 指中幼、晚幼嗜碱性粒细胞；^c^ 指早幼、中幼及晚幼红细胞

**表2:** 骨髓细胞识别谱

系统	系列	原始阶段	幼稚阶段	成熟阶段	异常形态细胞
应纳入类别					
粒细胞	中性粒细胞	原始粒细胞	早幼粒细胞、中性中幼粒细胞、中性晚幼粒细胞	中性杆状核粒细胞、中性分叶核粒细胞	异常早幼粒细胞、Pelger-Huët畸形粒细胞、多分叶核粒细胞
	嗜酸性粒细胞		嗜酸性中幼粒细胞、嗜酸性晚幼粒细胞	嗜酸性杆状核粒细胞、嗜酸性分叶核粒细胞	异常嗜酸性粒细胞
	嗜碱性粒细胞		嗜碱性中幼粒细胞、嗜碱性晚幼粒细胞	嗜碱性杆状核粒细胞、嗜碱性分叶核粒细胞	异常嗜碱性粒细胞
红细胞		原始红细胞	早幼红细胞、中幼红细胞、晚幼红细胞	成熟红细胞^a^	巨幼红细胞
巨核细胞		原始巨核细胞	幼稚巨核细胞	颗粒型巨核细胞、产板型巨核细胞、裸核巨核细胞、血小板	小巨核细胞
淋巴细胞		原始淋巴细胞	幼稚淋巴细胞	成熟淋巴细胞	异常淋巴细胞或不典型改变的淋巴细胞（怀疑肿瘤性）
单核细胞		原始单核细胞	幼稚单核细胞	成熟单核细胞	异常单核细胞或不典型改变的单核细胞
浆细胞		原始浆细胞	幼稚浆细胞	成熟浆细胞	巨大骨髓瘤细胞
其他细胞				肥大细胞、组织细胞、成骨细胞、破骨细胞	异常组织细胞
外来细胞					癌细胞团^b^
后期需纳入类别					
粒细胞					含Auer小体原始粒细胞、异常中幼粒细胞、其他异常形态粒细胞
红细胞					巨幼样变红细胞、多核红细胞、核间桥、核碎裂、花瓣核、母子核
巨核细胞					多核圆核巨核细胞、单圆核巨核细胞、多分叶核巨核细胞
淋巴细胞					各亚型异常淋巴细胞或不典型改变的淋巴细胞（怀疑肿瘤性）
浆细胞					Mott细胞、火焰状浆细胞、含包涵体浆细胞、含Dutcher小体浆细胞、其他形态异常浆细胞
其他细胞					不典型肥大细胞、噬血细胞、海蓝组织细胞、戈谢细胞、尼曼-匹克细胞、分类不明细胞、其他
外来细胞					腺癌细胞、鳞癌细胞、未分化癌细胞、神经母细胞瘤、肉瘤细胞

**注** ^a^ 骨髓像不标注识别成熟红细胞；^b^ 癌细胞团是识别转移癌的重要形态学特征，需要单列出来标注识别

（七）病理细胞的细分类

病理细胞的形态特征具有一定的复杂性，算法训练有一定难度，识别性能需要大样本、多中心评估验证。在初始训练人工智能辅助识别病理细胞时不建议采用笼统的细胞名称。病理意义相同的细胞通常是一大类细胞，它们的形态特征各异，细胞图像相似性差，识别准确率低。建议从细胞形态学的角度做细分类，如多毛细胞、花形核淋巴细胞、单核细胞样异型淋巴细胞，这样有益于提高人工智能识别细胞的准确率。最后，在报告中将细分类的数据归纳为大类细胞来报告，如异型淋巴细胞和异常淋巴细胞等。这样既提高了人工智能辅助细胞识别的准确率，又符合现有检验报告的细胞类别和名称的要求。

（八）细胞训练与测试的要求

训练集和测试集取样的病例数之和应大于1 000例，并来源于多家具有独立血液系统形态学诊断能力的医院或实验室，以保证样本来源和数量的多样性[52]。训练集包含的常见种类的细胞数应大于5 000个，少见种类细胞数应大于2 000个。细胞标注人员应具备医学检验技师及同等以上资质，并有5年以上血细胞形态学检查工作经历，判读标本总例数不低于10 000例。人工智能辅助血细胞识别的性能评估指标在不同类别的细胞中要求不同，常见细胞识别的准确率应大于95％，精确率大于90％，召回率大于95％[53]–[54]。少见或疑难细胞的判定指标应该在后期的训练中不断提高，以满足细胞形态学诊断的需求。人工智能出具的细胞判别结果应注明细胞类别及其权重比例，以帮助审核人员评估识别结果是否可靠、设备性能是否良好。

人工智能辅助血细胞识别的性能评价方案：至少选取200份制片染色良好的骨髓涂片或血涂片，使用人工智能辅助血细胞形态分析系统进行血细胞预分类，在每个骨髓涂片中采集并分析至少500个有核细胞，在每个血涂片中采集分析至少200个白细胞。同时由符合资质的2位检验医师/技师进行分类，对比系统预分类结果与检验医师/技师的分类结果，计算人工智能辅助血细胞识别的准确率、召回率和精确率。

三、人工智能辅助血细胞形态学检查的临床应用

（一）设立质量控制标准

为了使人工智能结果更加准确，血细胞形态学人工智能设备本地使用测试的各项指标达标后，使用者应设立室内质控标准，评判设备的细胞识别归类计数结果是否符合室内质控标准，并通过随机样本抽查和异常结果复检来评估结果的准确性和稳定性[54]。

1. 建立不合格标本的处理方法：使用者应评判血涂片与骨髓涂片的推片质量、染色质量是否合格。对于质量不合格的标本，应停止上机检测或放弃人工智能识别细胞的检测数据，重新制片染色后识别或直接人工镜检出具报告。

2. 设置异常比例提示与危急值预警：参考外周血和骨髓象分类计数参考区间，提示比例增高或减低；参考外周血分类计数指南，报告危急值[55]。对于骨髓分类比例，应注意原始细胞和异常细胞的比例，给予提示和建议。对于所有的细胞分类比例，应特别重视比例分布发生突兀变化的情况，鉴别其发生的缘由。

3. 人工智能设备的临床应用应从预分类逐步过渡到直接分类：鉴于人工智能辅助血细胞形态学检查处于临床应用的初期，使用者需要将人工智能的预分类结果与人工分类报告进行比对，以评估人工智能预分类的准确性。当设备使用成熟稳定、结果准确可靠时，经质控专家组审核分析后，可在一定的疾病范围内尝试把人工智能设备的分类结果逐步推广到临床应用。

（二）辅助人工出具血细胞形态学检查诊断报告

1. 报告内容与报告形式

（1）报告内容：内容常规包括基本信息、图片资料、细胞比例（正常与病理细胞）等。细胞类别及比例遵照现有的外周血和骨髓形态学人工显微镜手工分类报告标准[47]。例如在外周血报告中原始细胞不报告具体系别，中性幼稚粒细胞应划分阶段，异型（反应性）淋巴细胞及异常淋巴细胞不区分具体型别，但类似毛细胞、幼淋细胞（大细胞）等有显著形态学特征和疾病指向意义的细胞类别应在报告中描述说明。在骨髓报告中查见外来细胞的病例，应提示是否为转移癌细胞或淋巴系统的巨大瘤细胞等指向性信息，并做出相应的病理检查建议。

涂抹细胞增多有一定的临床诊断价值。由于制片等原因可能导致人工智能自动识别的白细胞总数中含有涂抹细胞，使自动分类结果不准确，因此白细胞比例计算时需要人工复核。当涂抹细胞>10％时，应进行单独提示。

当人工智能具备识别各类病理细胞能力时，可计算细分类的某类细胞占总类或某一大类细胞的比例，如幼淋细胞占淋巴细胞的比例、颗粒减少粒细胞占中性粒细胞的比例、发育异常的幼红细胞占幼红细胞的比例等。

（2）报告形式：血细胞形态学分类结果的报告形式推荐采用图文报告形式。图片储存区在界面上分类呈现各类单个细胞图片50～200张和（或）多细胞图片5～10张，以备观察分析和打印使用。

2. 人工审核

人工智能血细胞识别结果的审核应该是全方位的，包括患者的基本信息、项目名称、工作流程以及数据的准确性。检测数据的准确性可通过手工复核或结合MICM及其他检测结果综合评估判断。

关于人工智能细胞识别准确性的审核，可以在采集的真实视野下对无法分类的细胞和判读错误的细胞进行人工修正，也可以在归类后的细胞图片上对判读错误的细胞进行修改矫正。

在外周血细胞形态学检查诊断报告中，需要人工审核和添加血涂片的细胞特征和结果提示意见，包括涂片中白细胞总数情况和异常细胞特征，红细胞、白细胞、血小板的数量及形态，有无寄生虫等。在骨髓细胞形态学检查诊断报告中，由于骨髓中细胞类别多、形态变异复杂，特别是骨髓中的血液肿瘤细胞具有高度异质性，存在相同疾病伴有不同形态细胞以及相同形态细胞但不同疾病的问题。在人工智能无法识别或量化病理细胞的情况下，需要人工复核与观察分析各种细胞发育异常的现象并归纳报告，包括：①粒细胞系统：观察粒细胞的数量与形态，注意原始细胞比例、有无Auer小体、Pelger-Huët核畸形、粒细胞中毒性改变、粒细胞遗传性改变、粒细胞分叶过多或过少、颗粒减少、双核、巨幼变及巨幼样变等；②红细胞系统：观察红细胞的数量与形态，注意有无母子核、多核幼红细胞、双核、核间桥、核芽孢、巨幼变及巨幼样变等；③淋巴细胞系统：观察淋巴细胞的数量与形态，观察异常淋巴细胞中各种类型肿瘤细胞的形态特征和比例分布；④巨核细胞及血小板：观察巨核细胞的数量与形态，注意巨核细胞有无成熟障碍，有无小巨核细胞、圆核巨核细胞等以及血小板数量多少，有无血小板大小异常、有无血小板卫星现象；⑤其他有核细胞：注意有无异常浆细胞、异常巨核细胞、巨大淋巴瘤细胞、噬血细胞、转移癌细胞等；⑥其他情形：如白细胞数量太多导致细胞重叠仪器无法分类，白细胞分类结果与血液分析仪明显不符等。

细胞化学染色是支持形态学诊断结论的重要依据，也是判断和修正人工智能细胞识别比例的依据。建议人工智能分类结果在具有某种疾病病理改变指向的情况下，与人工分类操作一样，可结合细胞化学染色结果分析后再出具相关报告。同时结合患者的临床表现、影像检查及实验检查相关结果进行综合分析，为临床提供诊断意向和合理性的建议。

四、数据管理和信息安全

细胞病理数字图像的存储需要满足数据安全和高效管理的要求，同时方便接入医院信息系统、实验室信息管理系统、人工智能辅助诊断系统。具体要求如下：①受检者信息应能与图像、分类计数数据对应；②各数据集均须备份，在存储容量达到一定阈值时示警，以便系统管理员及时更新存储；③所有患者的基本信息、细胞数字图像信息以及分类计数的实验数据都是受检者真实诊疗活动的记录，数据存储安全与患者隐私都必须得到保护，防止泄露[56]–[58]；④管理数据访问权限，使用者需要登录账号进行身份验证，并保留操作日志，数据的传输和存储均需加密，以防止数据被旁路获取和篡改；⑤建议仅使用本软件计算分析。确实需要远端数据传输时，必须采用数据脱敏机制，以去除任何具备指向性的个人信息。

报告发出后，需要对涂片，包括采集的图像进行分类归档，妥善保管，保存时限按各实验室标准操作规程进行处置。一般外周血涂片标本保存一周（备复检），电子数据保存15年；骨髓涂片标本保存10年，电子数据保存30年[59]–[61]。
